# Restoring Age-Related Cognitive Decline through Environmental Enrichment: A Transcriptomic Approach

**DOI:** 10.3390/cells11233864

**Published:** 2022-11-30

**Authors:** Silvio Schmidt, Madlen Haase, Lena Best, Marco Groth, Julia Lindner, Otto W. Witte, Christoph Kaleta, Christiane Frahm

**Affiliations:** 1 Hans-Berger Department of Neurology, University Hospital Jena, Friedrich Schiller University Jena, D-07747 Jena, Germany; 2 Institute for Experimental Medicine, Kiel University, c/o Transfusion Medicine, University Medical Center Schleswig-Holstein, Campus Kiel, Michaelisstraße 5, Haus 17, D-24105 Kiel, Germany; 3 Core Facility DNA Sequencing, Leibniz Institute on Aging—Fritz Lipmann Institute, D-07745 Jena, Germany

**Keywords:** aging, brain, enriched environment, cognition, RNA sequencing

## Abstract

Cognitive decline is one of the greatest health threats of old age and the maintenance of optimal brain function across a lifespan remains a big challenge. The hippocampus is considered particularly vulnerable but there is cross-species consensus that its functional integrity benefits from the early and continuous exercise of demanding physical, social and mental activities, also referred to as environmental enrichment (EE). Here, we investigated the extent to which late-onset EE can improve the already-impaired cognitive abilities of lifelong deprived C57BL/6 mice and how it affects gene expression in the hippocampus. To this end, 5- and 24-month-old mice housed in standard cages (5mSC and 24mSC) and 24-month-old mice exposed to EE in the last 2 months of their life (24mEE) were subjected to a Barnes maze task followed by next-generation RNA sequencing of the hippocampal tissue. Our analyses showed that late-onset EE was able to restore deficits in spatial learning and short-term memory in 24-month-old mice. These positive cognitive effects were reflected by specific changes in the hippocampal transcriptome, where late-onset EE affected transcription much more than age (24mSC vs. 24mEE: 1311 DEGs, 24mSC vs. 5mSC: 860 DEGs). Remarkably, a small intersection of 72 age-related DEGs was counter-regulated by late-onset EE. Of these, Bcl3, Cttnbp2, Diexf, Esr2, Grb10, Il4ra, Inhba, Rras2, Rps6ka1 and Socs3 appear to be particularly relevant as key regulators involved in dendritic spine plasticity and in age-relevant molecular signaling cascades mediating senescence, insulin resistance, apoptosis and tissue regeneration. In summary, our observations suggest that the brains of aged mice in standard cage housing preserve a considerable degree of plasticity. Switching them to EE proved to be a promising and non-pharmacological intervention against cognitive decline.

## 1. Introduction

Worldwide, the population is aging. As more people live longer, the prevalence of age-related cognitive decline and neurodegenerative disorders will increase. The decline of cognitive functions is currently one of the greatest health threats of old age [1]. The hippocampus is engaged in various cognitive tasks in rodents as well as in humans, among them spatial learning, and was revealed as the most sensitive brain region involved in cognitive aging [2]. Functional imaging techniques detect age-related changes in the structure, metabolism and functional connectivity of the hippocampal formation, which potentially affect cognitive abilities [3,4,5]. On the molecular level, immune- and inflammation-related genes are activated during aging and predispose the brain to neurodegenerative processes [6,7,8,9]. There are promising studies on how the model organism makes modifications [10]. The environment and its stimulating aspects have profound effects on brain plasticity. An enriched environment (EE) is a paradigm, which provides mice with a broad spectrum of stimulation at the social, sensory, motor and cognitive levels. Exposure of rodents to EE changes brain morphology and physiology as well as cerebral molecular processes [11,12]. As a consequence EE leads to better motor and cognitive outcomes in animals with stroke or other brain pathophysiologies and protects from cognitive decline in aged rodents [11]. Additionally, in humans, early cognitive enrichment is advantageous for maintaining cognition over a lifespan [13]. Highly educated persons are protected in some aspects from age-related cognitive decline and from dementia, which is often postponed [13]. There are also beneficial effects when providing physical activity such as wheel running for mice or exercise in humans; however, especially at an older age, physical activity alone did not show a clear effect on cognition [14,15,16]. The identification of mechanisms mediating adaptive neurobiological plasticity to enhance the functional capacity of neural systems and to maintain optimal brain performance across a lifespan is still a big challenge [12]. However, as observed in both animal and human models, it is well known that dealing with a complex environment strengthens the bi-directional signaling of the environment and brain, builds cognitive reserve, and enhances the brain’s functional capacity [12]. 

Developing targeted therapeutic interventions to attenuate cognitive impairments in the elderly demands a greater understanding of the processes underlying normal brain aging and subsequently, it must be determined how age-related pathways can be modified. The aim of this study is to analyze the plasticity of aged brains with the subsequent characterization of their cognitive functions and identify the genes and pathways that modulate the brain following enriched activity. Specifically, we will study the influence of late-onset EE exposure on hippocampal aging and cognitive function in male C57BL/6 mice. The mice for EE were taken from standard sensory and activity-deprived housing and compared to groups kept in the same impoverished environment. Hippocampal function following EE will be assessed by a learning and memory test (Barnes maze). On the molecular level, RNA-seq data will be obtained and analyzed from the hippocampal tissue of mice of different ages (5 and 24 months old) and from 24-month-old mice who spent their last 2 months in an EE. By understanding the molecular mechanisms underlying the effects of enriched activity in animal models, the basis for identifying and influencing neural mechanisms will be established in order to specifically exploit the positive effects of physical and social activity for healthy aging and cognition in the elderly.

## 2. Materials & Methods

### 2.1. Animals

Male C57BL/6 mice were bred in the Central Experimental Animal Husbandry (ZET) at the University Hospital Jena, Germany. Mice were housed in groups in standard cages (EU Standard Type III 1290D, 820 cm^2^ 425 × 276 × 153 mm, Tecniplast, Italy) with access to food and water ad libitum. Enriched environment cages (EU Standard TYP IV S 1354G, 1820 cm^2^ 598 × 380 × 200 mm, Tecniplast, Italy) were equipped with two running wheels, climbing platforms, plastic tubes and tunnels, small houses and nesting materials. The positions of all objects were changed weekly. Mice were housed in groups of 6–10 animals.

All mice were randomly assigned to the treatment groups. The animals were subjected to strict hygienic controls according to official standards and veterinary regulations. Holding conditions: 21 ± 2 °C, humidity 55 ± 10%, 10–15× air changes, 14 h/10 h day/night, 100 lux, graded air pressure with ΔP 25 Pa per step: animal room > hall > changing room > General area. The hygiene status according to Federation of European Laboratory Animal Science Associations (FELASA) was recorded by regular deductions and documented by means of health certificates. All units were considered as specified pathogen-free. All animal experiments were carried out in strict accordance with the recommendations of the European Commission on the protection of animals used for scientific purposes and were approved by the local authority for regulating animal experimentation (Thueringer Landesamt für Lebensmittelsicherheit und Verbraucherschutz [TLLV], Germany). 

### 2.2. Barnes Maze Test

Mice at the age of 5 and 24 months housed under standard conditions (5mSC, n = 8 and 24mSC, n = 10) and 24-month-old mice which were exposed for their last 2 months to an EE (24mEE, n = 7, n = 6 for Retention test due to loss of one animal) were tested for their cognitive function by the Barnes maze test.

The maze consists of a circular platform, 90 cm in diameter with 20 evenly spaced holes in the periphery, each 5 cm in diameter. The platform was illuminated and mounted 1 m above the floor. Extra-maze cues for hippocampus-dependent learning were located on the walls for orientation. A single habituation trial was performed 1 day before the training started. The mouse was placed in the middle of the maze in a transparent start box. After 60 s, the box was lifted, and the mouse freely explored the maze for 120 s. Then, the mouse was guided to the escape hole connected to a box underneath the platform and remained there for 60 s. The next day, the 6-day training sessions with 3 trials per mouse started. The inter-trial interval was set to 15 min, during this time the maze was cleaned with 70% ethanol and rotated to avoid olfactory cues. The location of the target hole was unchanged for each mouse during the training sessions but was randomized across mice. On the first day, after the 6-day training session on day 7, the Probe trial was performed to test short-term memory. For the Probe trial, the escape box was removed and all holes were closed. The Retention test to test memory took place 7 days after the Probe trial on day 14, with the escape box set back in its original position. 

Probe trial and Retention test were each performed as a single 240 s trial. All experiments were recorded and mean velocity was automatically calculated by the video tracking system EthoVision^®^XT 6.1 software (Noldus, the Netherlands). Primary latency (first time to locate the target hole) was measured during the trial assessment. In addition, for the Probe trial, the success rate in finding the target hole was analyzed as well as the duration at the closed escape box. Specifically, the length of stay in the quadrant was measured; 1 hole to the right and 1 hole to the left of the original hole formed the boundary of this area. 

Statistical analysis was performed using a mixed ANOVA for the training period; for the individual test days, analysis was performed using a univariate ANOVA. Bonferroni correction was applied for multiple comparisons. Nominal data were analyzed using Pearson’s Chi-square test and Fisher’s exact method for post-hoc analysis [17] (IBM SPSS Statistics 27, Ehningen, Germany). Effect sizes were calculated in the form of mean differences [18].

### 2.3. Tissue and RNA Preparation

Another group of mice was maintained in 2 runs at different times with the same experimental EE setup and sacrificed by cervical dislocation following brain removal and hippocampal preparation (24mEE, 24mSC: n = 10 each, 5mSC: n = 8). The hippocampi were homogenized in 500 μL QIAzol Lysis Reagent (Qiagen GmbH, Hilden, Germany) and total RNA was isolated from each sample individually using the phenol/chloroform extraction method. RNA quantity and quality were determined spectrometrically by an ND-1000 (Nanodrop, Wilmington, DE, USA) and subsequently analyzed by an Agilent Bioanalyzer 2100 using the RNA 6000 nano kit (Agilent Technologies, Santa Clara, CA, USA). A RIN (RNA Integrity Number) value of greater than 7 was considered to be of good quality for sequencing. 

### 2.4. RNA-seq

Library preparation for individual samples and sequencing was performed using Illumina technology [19]. An amount of 2.5 μg of total RNA was used with Illumina’s TruSeq RNA sample prep kit v2 following the manufacturer’s instruction. Libraries were quality-checked and quantified using the Bioanalyzer 2100 and the DNA 7500 kit (Agilent). Sequencing was carried out by pooling 5 libraries per lane on a HiSeq2000/2500 in 50-cycle mode to obtain 50nt single-end reads. The reads were extracted in FASTQ format using bcl2fastq software tool (v1.8.2/v1.8.3) provided by Illumina resulting in ~35 Mio reads per sample.

### 2.5. Data Analysis

Mapping of the reads was performed using Tophat v1.4.1 [20], the mouse genome (mm10) and the UCSC [21] RefSeq annotation (version 2012-04-18) by allowing only reads mapping uniquely to the annotated regions. Based on the mapping results the reads per gene were counted using htseq-count of the HTSeq software package v0.5.4 [22] in union mode. This produced count data for 23.337 annotated genes for each sample.

Identification of differentially expressed genes (DEGs) was carried out based on the count data using the statistical package DESeq2 which tests for differential expression by use of negative binomial generalized linear models; using shrinkage estimation for dispersions and fold changes to improve stability and interpretability of estimates [23]. DEGs were obtained through pairwise comparisons between the treatment groups (young mice in standard cages vs. old mice in standard cages and old mice following EE vs. old mice from standard cages) while accounting for sequencing run. Obtained *p*-values were corrected for multiple testing using Benjamini–Hochberg [24]. Genes with a *p*-value (false discovery rate, FDR) <0.05 were regarded to be differentially expressed. Gene functional annotation was performed with the functional annotation tool DAVID Bioinformatics Resources (2021 Update) using the following threshold: count = 10–4, EASE = 0.1. Protein–protein association networks were constructed with stringDB version 11.5 (string-db.org accessed on 15 November 2021; PMID: 30476243). An interaction confidence score (based on experimental interactions and pathway databases) was calculated for the 72 counter-regulated genes and 50 first-shell interactors. Only input genes with at minimum 2 predicted interactions and confidence scores above 0.9 where visualized.

### 2.6. qPCR

qPCR analysis was applied to verify the expression of 16 selected genes. Total RNA (right hippocampus) taken from the same batch and the same groups of animals as for sequencing (24mEE, 24mSC: n = 10 each, 5mSC: n = 8) was transcribed into cDNA (RevertAid First Strand cDNA Synthesis Kit, Thermo Fisher Scientific, Waltham, MA, USA). The qPCR was performed with Brilliant III SYBR^®^Green QPCR Master Mix (Agilent Technologies, Santa Clara, CA, USA) and specific mouse primers (biomers.net GmbH, Ulm, Germany, Supplementary Table S4). Amplification was performed using Rotor-Gene 6000 cycler (Qiagen GmbH) applying the following cycle conditions: 3 min polymerase activation (95 °C) followed by 40 amplification cycles (95 °C for 10 s, 60 °C for 15 s). In a previous study, we confirmed Gapdh and Hmbs as suitable housekeeping genes in aged mice [25]. The mRNA transcript ratios were calculated using the Pfaffl equation [26].

## 3. Results

### 3.1. Performance in the Barnes Maze Test over the Training Days

**Figure 1:**
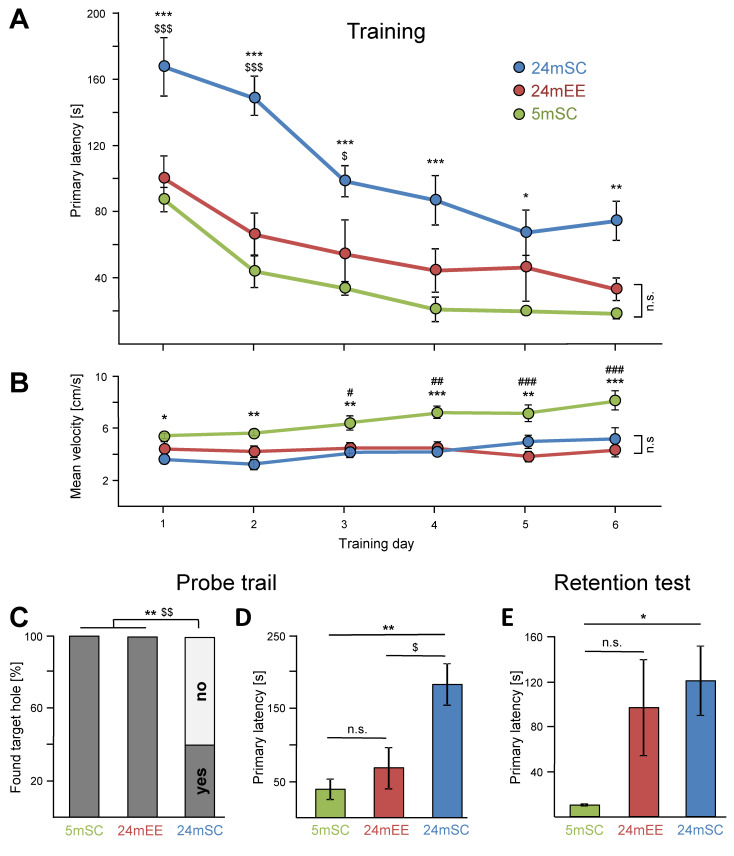
**Late-onset EE restores deficits in spatial learning and short-term memory in 24-month-old mice in the Barnes maze test.** (**A**) Improvement over the training period was observed for all groups (F_5,110_ = 26.44, *p <* 0.001). Under standard housing conditions, 24-month-old mice (24mSC, n = 10) took significantly more time to reach the escape hole (primary latency) compared to 5-month-old mice (5mSC, n = 8) on each training day. After late-onset EE exposure, 24-month-old mice (24mEE, n = 7) performed better than 24mSC mice on days 1, 2 and 3 and notably were not significantly different from 5mSC (F_2,22_ = 18.48, *p <* 0.001). (**B**) 5-month-old mice (5mSC) were significantly faster (mean velocity) than 24-month-old mice (24mSC and 24mEE) but there was no significant difference between the old mice groups (F_2,22_ = 16.46; *p <* 0.001). (**C**–**E**) Significant effects of aging and late-onset EE were also evident during the Probe trial (F_2,22_ = 9.55, *p* = 0.001) and the Retention test (F_2,21_ = 4.27, *p* = 0.028). All parameters are shown as mean ± SEM, success rate in finding the target hole is presented as %. Retention test in the 24mEE group was performed with n = 6, due to loss of one animal. Training period was tested with mixed ANOVA, Probe trial and Retention test were analyzed with univariate ANOVA, success rate was tested with Fisher’s exact test, * = 5mSC vs. 24mSC, # = 5mSC vs. 24mEE, $ = 24mSC vs. 24mEE, *, $, # *p* ≤ 0.05, **, $$, ## *p* ≤ 0.01, ***, $$$, ### *p* ≤ 0.001.

After 8 weeks of exposure to an enriched environment or housing under standard conditions, animals were subjected to the Barnes maze to test their spatial learning and memory. During the test phase, the mice remained under their respective housing conditions. The time to identify the target hole (primary latency) was significantly decreased with increased training time in young (5mSC) and aged controls housed in standard conditions (24mSC), as well as in aged mice exposed to EE (24mEE mice) (mixed ANOVA, effect of training day: F_5,110_ = 26.44, *p <* 0.001, 5mSC: F_5,110_ = 6.82, *p <* 0.001, 24mSC: F_5,110_ = 20.76, *p <* 0.001, 24mEE: F _5,110_ = 4.78, *p* = 0.001) (Figure 1A). The primary latency of the 5mSC mice was significantly shorter on the training days 2 (*p* = 0.015), 3 (*p* = 0.001), 4 (*p <* 0.001), 5 (*p <* 0.001) and 6 (*p <* 0.001) compared to the first training day. The 24mSC and 24mEE mice showed an improvement in performance over time on days 3 (24mSC: *p <* 0.001; 24mEE: *p* = 0.017), 4 (24mSC: *p <* 0.001; 24mEE: *p* = 0.002), 5 (24mSC: *p <* 0.001; 24mEE: *p* = 0.003) and 6 (24mSC: *p <* 0.001; 24mEE: *p <* 0.001) vs. day 1. Mean velocity became progressively faster in the 5mSC and 24mSC over the training period, while the 24mEE mice showed no significant improvement over the training period (mixed ANOVA, effect of training day: F_5,110_ = 6.88, *p <* 0.001, 5mSC: F _5,110_ = 6.02, *p <* 0.001, 24mSC: F_5,110_ = 5.29, *p <* 0.001) (Figure 1B). The 5mSC mice showed higher mean velocity on days 4 (*p* = 0.017), 5 (*p* = 0.022) and 6 (*p <* 0.001) compared to the first training day and the 24mSC mice were better on days 5 (*p* = 0.050) and 6 (*p* = 0.001) compared to the first training day. The measured values, as well as the effect sizes in the form of mean differences, are shown in Supplementary Table S5.

Altogether all groups were able to improve their performance in the Barnes maze test over the training period, independent of age and housing conditions. 

### 3.2. Enriched Environment Improved Barnes Maze Performance in Aged Mice

When comparing the primary latency of all three groups (5mSC, 24mSC, 24mEE), a significant effect was determined (mixed ANOVA, effect of group: F_2,22_ = 18.48, *p <* 0.001). When comparing aged and young mice during training, a significant learning deficit was observed in 24mSC compared to 5mSC throughout the training period and on each training day (days 1 and 2: *p <* 0.001, days 3 and 4: *p* = 0.001, day 5: *p* = 0.022, day 6: *p* = 0.005, all days together: *p <* 0.001). After EE exposure, the 24mEE mice performed significantly better than their age-matched controls (24mSC) at days 1 (*p* = 0.001), 2 (*p <* 0.001) and 3 (*p* = 0.048) and were also significantly better when taking all training days together (*p* = 0.002). Noteworthily, the 24mEE mice did not show a difference in the primary latency compared to 5mSC on any tested day (Figure 1A). Mean velocity was measured on each of the 6 training days. On all days, 24mSC were significantly slower than 5mSC (day 1: *p* = 0.016, day 2: *p* = 0.002, day 3: *p* = 0.008, day 4 and day 6: *p* = 0.001, day 5: *p* = 0.004). The 24mEE mice were significantly slower than the 5mSC mice on day 3 (*p* = 0.047), day 4 (*p* = 0.002) and on days 5 and 6 (*p <* 0.001). Over the entire training period, the 24mSC and 24mEE mice were significantly slower than the 5mSC mice but no significant difference was found between 24mSC and 24mEE (mixed ANOVA, effect of group: F_2,22_ = 16.46; *p <* 0.001). This rules out that the better cognitive performance of the 24mEE mice in the Barnes maze test is partly due to a higher mean velocity (Figure 1B).

On day 7, the Probe trial was performed to test short-term memory. All holes were closed and the percentage of mice that found the target (Figure 1C), the time to reach the original escape hole (primary latency) (Figure 1D), as well as the time the mice stayed at the closed original target hole (Figure 1D), were measured. The original hole, now closed, was found by both the 5mSC and the 24mEE mice, while 60% of the old 24mSC mice did not find the hole (Pearson’s Chi-square test with Fisher’s exact approach to post-hoc analysis [17], Figure 1C). The 24mSC mice required more time and showed significantly increased primary latency to the original target compared to the 5mSC (*p* = 0.001) and 24mEE mice (*p* = 0.014). Remarkably, the primary latency of the 24mEE mice did not differ from that of the 5mSC mice (univariate ANOVA, group effect: F_2,22_ = 9.55, *p* = 0.001) (Figure 1D). The analysis of mean velocity revealed significant differences between age groups but no difference between the housing of the 24-month-old mice (univariate ANOVA, group effect: F_2,22_ = 18.09, *p <* 0.001, 24mSC vs. 5mSC *p <* 0.001, 24mEE vs. 5mSC *p* = 0.003, 24mEE vs. 24mSC n.s.). The measured velocities are as follows: 5mSC: 3.9 ± 0.4 s, 24mSC: 1.3 ± 0.2, 24mEE: 2.1 ± 0.4 s.

Long-term memory was tested in the Retention test on day 15. The 5mSC mice found the original target hole faster than the 24mSC mice (univariate ANOVA, group effect: F_2,21_ = 4.27, *p* = 0.028, 5mSC vs. 24mSC *p* = 0.029). EE did not shorten primary latency and thus has no effect on long-term memory (Figure 1E). According to the Probe trial, the mean velocity did not differ between the 24-month age groups but a significant difference was shown in the 5mSC group (univariate ANOVA, F_2,21_ = 29.78, *p <* 0.001; 5mSC vs. 24mEE and 24mSC *p <* 0.001, 24mEE vs. 24mSC n.s.). The measured mean velocities are as follows: 5mSC: 6.7 ± 0.2 s, 24mSC 2.00 ± 0.5 s, 24mEE 2.9 ± 0.7. The measured values, as well as the effect sizes in the form of mean differences, are shown in Supplementary Table S5.

### 3.3. Aging of the Hippocampus Changes Gene Expression

**Figure 2:**
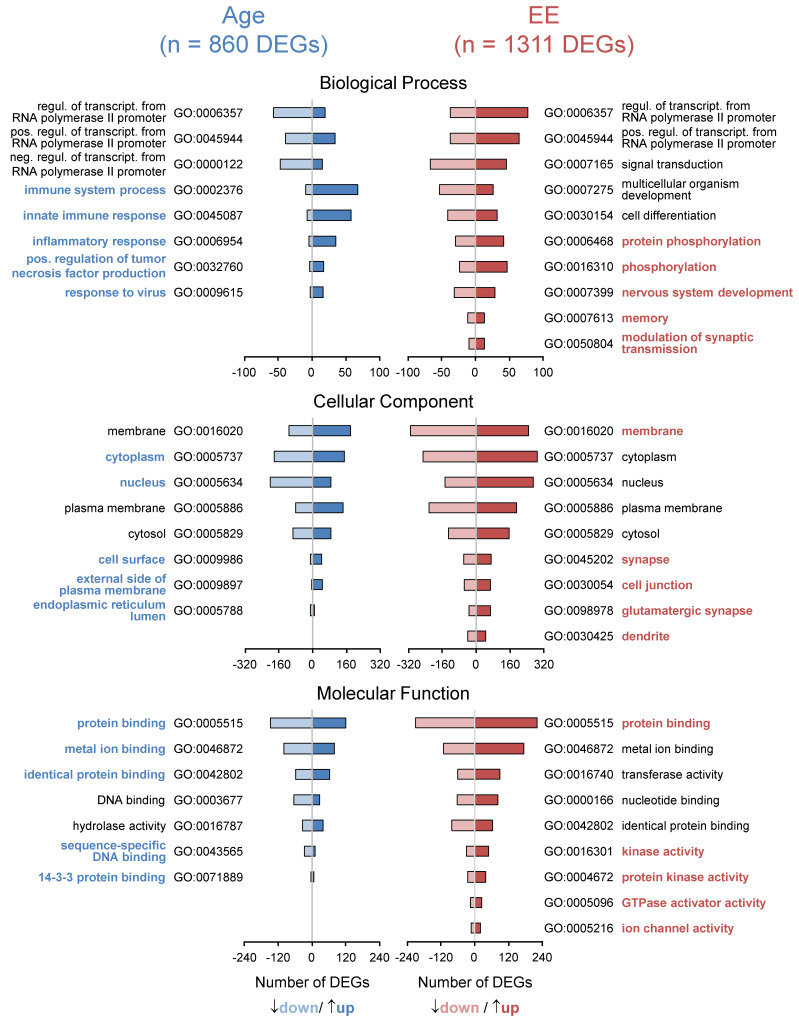
**Late-onset EE affects hippocampal gene expression more than age.** Aging in standard housing conditions revealed n = 860 DEGs (5mSC vs. 24mSC, left side in blue) whereas late-onset EE revealed n = 1311 DEGs (24mSC vs. 24mEE, right side in red). For both sets of DEGs, functional annotation considering the DAVID gene ontology categories “biological process”, “cellular component” and “molecular function” was performed (threshold: count = 10–4, EASE = 0.1, *p <* 0.01), whereas for each category the intersection of the 5 most significant (colored and bold) and 5 GO-terms with most genes was selected for presentation. DEGs were obtained through pairwise comparisons between the treatment groups (5mSC: n = 8, 24mSC: n = 10, 24mEE: n = 10). The full set of DEGs and functional annotations including statistics are shown in Supplementary Tables S1 and S2.

We determined age-related transcriptional changes in the mice kept under standard conditions by comparing 24mSC vs. 5mSC and identified 860 age-related DEGs (430 up- and 430 down-regulated). Functional annotation of these genes was performed with the functional annotation tool DAVID Bioinformatics Resources (2021 Update, Figure 2). By sorting the 826 out of 860 genes that could be mapped to the ENSEMBL gene, as well as transcript ID and considering the gene ontology category “biological process” and given 10 counts as a minimum, the five most significant processes out of sixty-eight functional annotation chart records belong to the immune system process (GO:0002376), innate immune response (GO:0045087), inflammatory response (GO:0006954), response to viruses (GO:0009615) and positive regulation of tumor necrosis factor production (GO:0032760). The five charts with the most genes involved are: immune system process (GO:0002376), regulation of transcription from RNA polymerase II promoter (GO:0006357), positive regulation of transcription from RNA polymerase II promoter (GO:0045944), innate immune response (GO:0045087) and negative regulation of transcription from RNA polymerase II promoter (GO:0000122). By sorting the genes considering the gene ontology category “cellular compartments”, the five most significant out of thirty GO terms are: cytoplasm (GO:0005737), external side of the plasma membrane (GO:0009897), endoplasmic reticulum lumen (GO:0005788), nucleus (GO:0005634) and cell surface (GO:0009986). The five charts with the most genes involved are: cytoplasm (GO:0005737), nucleus (GO:0005634), membrane (GO:0016020), plasma membrane (GO:0005886) and cytosol (GO:0005829). By sorting the genes into “molecular functions”, the five most significant out of twenty-six charts all belong to binding: protein binding (GO:0005515), identical protein binding (GO:0042802), metal ion binding (GO:0046872), sequence-specific DNA binding (GO:0043565) and 14-3-3 protein binding (GO:0071889). The five charts with the most genes involved are: protein binding (GO:0005515), metal ion binding (GO:0046872), identical protein binding (GO:0042802), DNA binding (GO:0003677) and hydrolase activity (GO:0016787). Age-related DEGs, as well as functional annotations, are shown in Supplementary Table S1.

### 3.4. Enriched Environment Changes Gene Expression in the Hippocampus

Following a 2-month housing of 22-month-old mice in an enriched environment, 1311 genes were found to be differentially regulated (24mEE vs. 24mSC: 669 up- and 642 down-regulated) in the hippocampus. These are more genes than are affected by the aging process and show that EE has a massive impact on the brain even in old mice (see Supplementary Table S2). 

Similar to the functional annotation of age-related genes, we processed these 1311 EE-regulated genes with the functional annotation tool DAVID Bioinformatics Resources (2021 Update, Figure 2). The five most significant “biological processes” out of one-hundred and one processes are: nervous system development (GO:0007399), protein phosphorylation (GO:0006468), phosphorylation (GO:0016310), modulation of chemical synaptic transmission (GO:0050804) and memory (GO:0007613). The five charts with the most genes involved are: regulation of transcription from RNA polymerase II promoter (GO:0006357), signal transduction (GO:0007165), positive regulation of transcription from RNA polymerase II promoter (GO:0045944), multicellular organism development (GO:0007275) and cell differentiation (GO:0030154). The five most significant processes out of eighty-three belonging to the “cellular component” are: glutamatergic synapse (GO:0098978), membrane (GO:0016020), synapse (GO:0045202), cell junction (GO:0030054) and dendrite (GO:0030425). The five charts with the most genes involved are: membrane (GO:0016020), cytoplasm (GO:0005737), nucleus (GO:0005634), plasma membrane (GO:0005886) and cytosol (GO:0005829). With respect to “molecular function”, the five most significant processes out of sixty are: protein binding (GO:0005515), ion channel activity (GO:0005216), protein kinase activity (GO:0004672), kinase activity (GO:0016301) and GTPase activator activity (GO:0005096). The five charts with the most gene counts are: protein binding (GO:0005515), metal ion binding (GO:0046872), transferase activity (GO:0016740), nucleotide binding (GO:0000166) and identical protein binding (GO:0042802). EE-related DEGs, as well as functional annotations, are shown in Supplementary Table S2. 

### 3.5. Enriched Environment Reverses Age-Related Gene Expression in the Hippocampus

**Figure 3:**
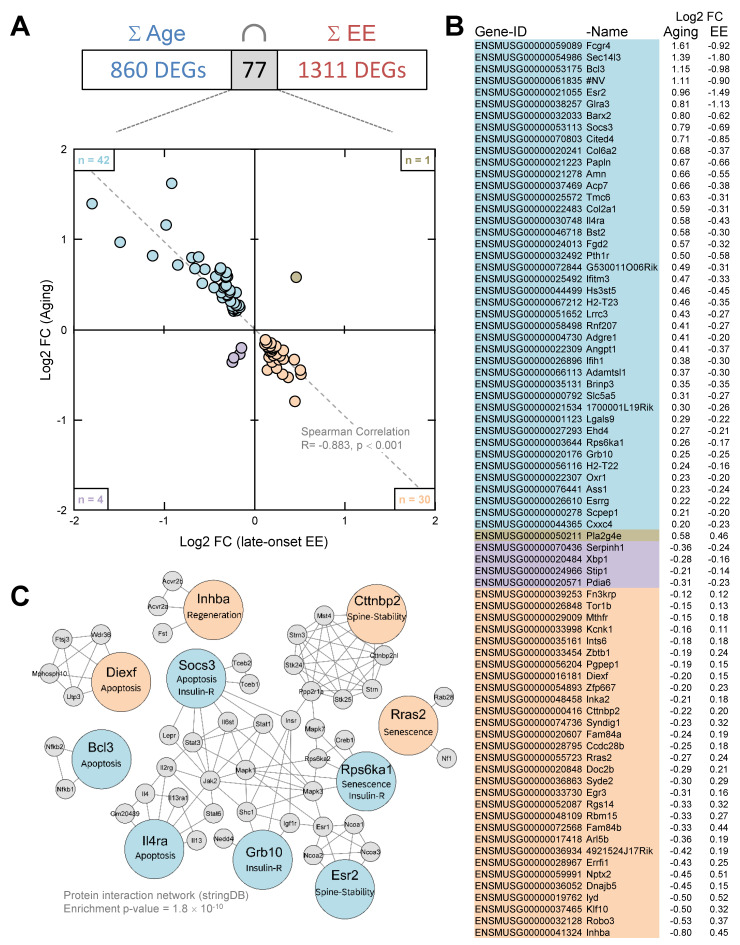
**Late-onset EE partially reverses age-related hippocampal gene expression changes.** (**A**) A small intersection of n = 77 age-related DEGs was affected by late-onset EE. The scatter plot (upper left and lower right quadrant), as well as the 12th panel in (**B**), shows among them n = 72 DEGs with counter-regulation. (**C**) StringDB association network identified 10 age-related DEGs as key regulators. Reverse-regulated genes (colored big circles) with at least 2 predicted interaction partners in the first shell (grey small circles, not included in our data set) and confidence scores > 0.9 are visualized. Additionally, their literature-based participation in known aging-relevant signaling cascades is indicated (see discussion for detailed descriptions). DEGs were obtained through pairwise comparisons between the treatment groups. Age: 5mSC (n = 8) vs. 24mSC (n = 10), EE: 24mSC (n = 10) vs. 24mEE (n = 10). The full set of DEGs, their functional annotations including statistics and the complete list of interactions is shown in Supplementary Table S3.

At the transcript level, 860 DEGs were identified during aging and 1311 DEGs following EE in old mice. In total, 77 out of the 860 age-related DEGs were affected by aging as well as following EE. A total of 42 out of 77 genes are up-regulated by aging and down-regulated following EE, and 30 out of 77 genes are down-regulated by aging and up-regulated following EE. This means that 94% (72 out of 77) of the age-related hippocampal genes were counter-regulated following EE (Spearman correlation: R = −0.883, *p <* 0.001, Figure 3A,B). The expression changes detected by RNA-seq that were additionally validated on 16 out of the 77 DEGs by qPCR and Spearman correlation coefficients were calculated (RNA-seq: R = −0.982, *p <* 0.01; qPCR: R = −0.839, *p <* 0.01; see Supplementary Table S4). Functional annotation (“biological process”) of the 77 DEGs affected by aging (76 mapped to ENSEMBL ID) as well as following EE with a minimum of four genes per process revealed five out of seven processes in the following order of significance: immune system process (GO:0002376), response to viruses (GO:0009615), extracellular matrix organization (GO:0030198), positive regulation of transcription from RNA polymerase II promoter (GO:0045944) and negative regulation of apoptotic process (GO:0043066). The five charts with the most genes are: positive regulation of transcription from RNA polymerase II promoter (GO:0045944), immune system process (GO:0002376), negative regulation of apoptotic process (GO:0043066), positive regulation of transcription, DNA-templated (GO:0045893) and response to viruses (GO:0009615). The five most significant processes out of 14 for the “cellular component” are: early endosome membrane (GO:0031901), endosome (GO:0005768), cytoplasm (GO:0005737), synapse (GO:0045202) and plasma membrane (GO:0005886). The five charts with the most genes are: cytoplasm (GO:0005737), nucleus (GO:0005634), plasma membrane (GO:0005886), extracellular region (GO:0005576) and extracellular space (GO:0005615). For “molecular function” five processes could be identified. These are, in order of significance: signaling receptor binding (GO:0005102), identical protein binding (GO:0042802), transmembrane signaling receptor activity (GO:0004888), protein binding (GO:0005515) and sequence-specific DNA binding (GO:0043565). DEGs, as well as functional annotations, are shown in Supplementary Table S3. Additionally, by using stringDB, protein–protein associations were analyzed for the 72 counter-regulated genes to evaluate their predicted impact on the whole proteome. Based on experimental evidence and database annotations, for 16 genes as a minimum, one interaction was found to reach a confidence score of > 0.9. With at least two interactions, Socs3 (suppressor of cytokine signaling 3), Cttnbp2 (Cortactin-binding protein 2), Il4ra (Interleukin-4 Receptor Subunit Alpha), Rps6ka1 (Ribosomal protein S6 kinase alpha-1), Diexf (digestive organ expansion factor), Esr2 (estrogen receptor ß), Grb10 (Growth-factor receptor bound protein 10), Inhba (inhibin subunit beta A), Rras2 (Ras-related protein R-Ras2) and Bcl3 (B-cell lymphoma 3-encoded protein) were identified as interaction hubs in the protein–protein interaction network (Figure 3C, see Supplementary Table S3 for the complete list of interactions). The protein–protein interaction network showed significantly more interactions than would be expected by chance (enrichment *p*-value = 1.8 × 10^−10^).

## 4. Discussion

### 4.1. Enriched Environment Improves Cognitive Function in Aging

The beneficial effects of housing mice in an enriched environment on brain plasticity and cognitive and motor functions, including protection against psychiatric disorders and neurodegenerative processes, are well known [27,28,29]. At the cellular level, EE promotes the development of new neurons, increases dendritic branching, spine density and synaptogenesis and leads to a cascade of molecular and metabolic changes [30]. Other studies highlight the effect of EE on cognitive brain reserve [31]. The concept of cognitive reserve suggests that environmental stimuli can increase resilience even in cognitive aging. 

Here, in contrast to studies applying long-lasting EE [32,33], we tested whether an intervention starting late in life when cognitive impairment is already present [2], can still mediate a positive effect. To that end, we placed 22-month-old mice for 2 months in an EE with running wheels. The Barnes maze test was performed at 24 months of age to analyze learning and memory parameters. First, we determined the age effect and, as expected, 24-month-old mice showed a significant learning and memory deficit compared to 5-month-old mice. The age-related cognitive decline was evident during the learning phase, in the short- and long-term memory tests (Figure 1A–E). The young mice differed in their mean velocity compared to the old mice. They were significantly faster (Figure 1B). In a recent study published by our own group, we tested 3-, 9-, 15-, 24-, and 28-month-old mice in the Barnes maze test. The 24-month-old age group was statistically different from all younger groups in training time, confirming the results of the current study. In the previously published study, we were able to ascertain the impairments in cognitive function between 15 and 24 months [34]. When comparing 4–6-month- and 22–24-month-old C57BL/6Nia mice in the Morris water maze task (MWM), an improvement over time in both groups and an age-dependent difference in escape latency were observed during the training phase [2]. In another study with extensive testing, it was found that 24-month-old male C57BL/6J wild-type mice showed cognitive impairments in the MWM, Y-maze, and novel object recognition tests, as well as depressed and anxious behaviors in the tail suspension, forced swimming, open field, and elevated plus maze tests compared with 2-month-old mice [35]. In conclusion, our test results regarding age-related cognitive impairments are consistent with previously published data.

Comparing the 24mEE with the 24mSC mice, EE showed a strong positive effect on learning during training on short-term but not on long-term memory. The 24mEE mice improved significantly over the 24mSC mice; moreover, even to the point that they no longer differed significantly from the 5mSC mice in their cognitive performance. This was true for the primary latency during training and the Probe trial, for the ability to find the target hole, and for the time in the target quadrant. The measurement of mean velocity rules out the possibility that the better performance of the 24mEE mice was partially due to better motor functions (Figure 1B).

It has already been shown that long-lasting or lifelong housing in an enriched environment protects against cognitive decline up to a certain age. Water maze tests using 20-month-old female albino Swiss mice maintained from weaning under enriched conditions showed increased learning ability in finding the platform position and improved search strategy compared to age-matched controls [36]. Fuchs et al. showed that female Long-Evans rats, which were housed under an EE for up to 24 months, were better in the MWM learning test (successful trials) and memory Probe trial compared to rats that stayed in an EE only for a period of 18 to 24 months. Both conditions, the long-lasting and the later-started 4-month EE housing had positive effects, specifically on memory compared to standard housing. The effects on memory were greater with long-lasting EE but the enriched housing from 18 to 24 months also had positive effects [37].

We could show here that a late-onset EE in 22-month-old mice, at a time when cognitive deficits are already detectable [2,38], surprisingly still leads to positive effects in cognitive function. Under our Barnes maze testing conditions, we demonstrated an improvement in both learning and short-term memory, even to the extent that the 24mEE group did not differ significantly from the young 5mSC mice. These results impressively confirm that very old brains still respond positively to a complex environment, with physical activity certainly playing a major role. In a recently published study, we showed that voluntary running on a wheel for 2 months has a positive effect on cognition in 20-month-old mice [34].

### 4.2. Age-Related Gene Expression Changes in the Hippocampus

The hippocampus was chosen due to its central role in the development of age-related cognitive changes in mice and in humans [39,40]. First, we identified 860 age-regulated DEGs (24mSC vs. 5mSC). Half of the 860 genes were up-regulated and the other half were down-regulated (n = 430 each). The 860 DEGs were analyzed using the functional annotation tool DAVID Bioinformatics Resources (2021 Update). Age-regulated DEGs and their main functions were in agreement with previous results [41,42,43] and external publications [44,45] and identified immune process and transcriptional regulations, inflammation, and cytokine production as regulators in brain aging. Consistent with this, marker genes indicative of activated astrocytes and microglial cells were found to be more highly expressed in the old brain [39,46]. Li and colleagues identified 1054 DEGs in the hippocampus of 24- vs. 2-month-old male C57BL/6J mice that are closely related to neuroinflammation and involved in the regulation of immune processes, confirming our analysis [35]. Wang and colleagues analyzed the intersection of 139 up-regulated genes in the hippocampus of 24- and 29-month-old male C57BL/6 mice compared with 3-month-old mice and detected similar processes to our analysis: immune system process, innate immune response, defense response to viruses, and inflammatory response [44]. A cell type-specific analysis in the hippocampus is certainly worthwhile, as such an analysis in the whole brain has shown that aging triggers specific transcriptional processes in different cell types and that individual genes can be oppositely regulated between different cell types [47]. Some studies were also performed in rats and mice, combining the MWM to detect age-related memory impairment with downstream microarrays to identify genes and signaling pathways that may be associated with cognitive decline [48]: Male Fischer rats at 4, 14, and 24 months of age underwent MWM testing and the older rats showed a significant reduction in memory performance compared to the other two age groups. Microarray analysis of the CA1 region of the hippocampus revealed known age-related processes, including inflammation, oxidative stress, altered protein processing, and decreased mitochondrial function. Newly identified processes included down-regulated early response signaling, biosynthesis, and activity-regulated synaptogenesis, as well as up-regulated myelin turnover, cholesterol synthesis, lipid and monoamine metabolism, iron utilization, structural reorganization, intracellular Ca2+ release pathways, transcriptional regulators, and cytokines. Verbitsky and colleagues have already identified a specific deficit in spatial memory in 15-month-old C57BL/6NIA mice using their MWM testing paradigm. Using microarrays, altered expression of genes related to inflammation, protein processing, and oxidative stress was detected in the hippocampus, as well as an alteration in synaptic plasticity processes [49].

### 4.3. Enriched Environment-Induced Gene Expression Changes in the Hippocampus

Next, we analyzed genes that were influenced by late-onset EE in old animals (24mEE vs. 24mSC). Knowledge about the effects of short-term EE on the hippocampal transcriptome is limited to young or middle-aged animals. By using high-density oligonucleotide microarrays and cortical samples, Rampon et al. [50] showed that a two-week-lasting EE in 4-month-old CBA/B6 hybrid mice leads to changes in the expression of around 100 genes that could be linked to neuronal structure and synaptic plasticity. In 3-month-old CD1 mice, RNA-seq-based analyses revealed that one-month-lasting EE regulates around 100 genes mainly involved in extracellular matrix modifications [51]. In middle-aged rodents, preselected gene sets were analyzed and only minor effects were revealed, leading to the conclusion that gene expression in the hippocampus is little affected by late-onset EE [52,53,54,55,56]. However, Park et al. [57] showed that the transcriptional effects of late-onset EE in middle-aged C57Bl/6J male mice even exceed those of young ones by targeting mainly neurodegenerative and senescence-associated pathways. Recently, late-onset EE in middle-aged female C57BL/6JR mice was shown to have greater effects on the translatom compared with lifelong EE [58], consistent with the recovery of age-related abnormal DNA methylation patterns in a comparable EE paradigm [59]. Our results provide further evidence/support that brain–environment interventions require a genetic molecular mechanism for cognitive changes to occur. We identified 1311 hippocampal DEGs after late-onset EE in 24-month-old C57Bl/6 mice. This high number exceeds the amount of 860 age-related DEGs and underscores the formidable plasticity of the aging brain. Gene functional annotation revealed comprehensive effects on all cellular compartments including the nucleus, the cytoplasm and membranes but most importantly on dendrites, cell junctions and synapses as main locations of neuronal network plasticity. Importantly, DEGs following EE are enriched in biological processes that mediate modulation of synaptic transmission, consistent with electrophysiological studies showing EE-related effects on both hippocampal neuronal excitability [60,61] and restoration of age-related deficits in synaptic LTP [62,63]. All these processes are known to be critical for the performance of hippocampus-dependent memory tasks [64,65] and there are well-founded indications for homeostatic synaptic scaling that mediates widespread adjustments in neuronal network connectivity and finally results in greater sparsity in the higher-order sub-network of place cell representations [66]. This increased sparsity in the hippocampal representation of space is believed to mediate the improvements in spatial learning and memory after exposure of animals to an EE [67]. In support of this, we revealed the enrichment of DEGs related to molecular mechanisms of phosphorylation such as protein phosphorylation, kinase activity, protein kinase activity and GTPase activator activity. In particular, post-transcriptional modifications mediated by the genes underlying these pathways represent ideal mechanisms to drive global network reconfiguration through homeostatic synaptic scaling response [68,69] and there are indications for their critical involvement in mediating the positive effects of late-onset EE on cognitive performance in middle-aged C57Bl/6J male mice [57].

### 4.4. Reversal of Age-Related Gene Expression Changes

We identified 77 age-regulated DEGs that were sensitive to late-onset EE, including 72 DEGs with counter-regulation. This suggests that some age-related changes at the transcriptional level are reversible by late-onset EE in old mice. The extent of the reversal found here (72 of 860 age-related DEGs; 8.4%) is in line with exercise-induced transcriptional reversal found in 18-month-old BALB/c mice (reversal of 117 of 1193 age-related DEGs; 9.8%) by Kohman et al. [70]. StringDB-based protein network analysis revealed 10 counter-regulated genes exhibiting an enrichment for predicted protein interactions. This suggests a strong functional link between genes counter-regulated by late-onset EE and may be related to the cognitive improvements in our tests or the overall positive, health-prolonging effects of EE. Hippocampal Esr2 interacts with the level of brain estrogen and is known to influence age-related cognitive decline in several ways [71]. Especially brain-derived estrogen represents a master regulator of the central metabolic state [72] and mediates signaling cascades affecting synaptic plasticity in males and females [73]. Increased Esr2 expression, as found here in aged mice, correlates with decreased formation of dendritic spines, whereas spine density recovers upon Esr2 expression declines [74]. Cttnbp2 is a neuron-specific regulator of the cytoskeleton highly enriched in dendritic spines and its experimental down-regulation reduces their formation, remodeling and size [75]. Its deficiency, as demonstrated here in old animals, is associated with neurodegenerative processes in PD [76] and autism [77]. Therefore, EE-induced reversed expression of Cttnbp2 and Esr2 is consistent with cognitive improvements and hippocampal spine-pool recovery as recently shown in senescent SAMP8 mice [78]. The GTPase Rras2 is essential for oligodendrocyte differentiation and survival and instrumental in the formation of myelin sheaths; experimental down-regulation leads to axonal degeneration in mice [79,80]. Depletion or reduced expression of Rras2, as shown here in aged mice, promotes cells to switch into senescence [81]. There is ample evidence that post-mitotic cellular senescence is a major feature of aging and that aging is thus at least partially programmed [82]. Accordingly, Rras2 rescue reverses senescent phenotypes [81], which may be initiated by our experimental EE paradigm. In line with this, aging-related up-regulation of Rps6ka1, a hub gene in the senescent SAMP8 mice [83], is associated with Alzheimer’s disease development, and experimental down-regulation (as here demonstrated by late-onset EE), ameliorates associated deficits in synaptic plasticity and spatial memory [84] and increases the lifespan of S6K1−/− mice [85]. Beyond reversing senescent phenotypes, down-regulation of Rps6ka1 also restores deficient insulin sensitivity in aged S6K1−/− mice [86]. This is an important observation as the progression of central insulin resistance renders cells insensitive to external inputs and accelerates cognitive decline, neurodegenerative disease and aging of the brain [87,88,89]. In addition to Rps6ka1 expression changes, we found further evidence that the insulin-dependent aspect of aging can be positively influenced by late-onset EE. Age-related increased expression of Grb10, a known inhibitor of insulin signaling and a negative regulator of neuronal growth and commitment [90], was reversed by late-onset EE. In line with our findings, down-regulation of Grb10 in the hippocampus using short hairpin RNA was shown to be neuroprotective and restored impaired memory and learning abilities in insulin-resistant diabetic rats [91]. SOCS3 also mediates insulin resistance and elevated SOCS3 levels, as shown here in aged mice, may predispose to neurodegenerative diseases [92]. Additionally noteworthy is the observation that suppression of increased SOCS3 expression after spinal cord injuries protects against apoptosis and finally facilitates their survival and regeneration [93,94,95,96]. There is accumulating evidence that increased susceptibility to apoptosis contributes to aging of the brain [97] by reducing the number of stressed but functional cells [98]. Reduced apoptosis in association with EE was found in young [99], aged [100] and senescent SAMP8 mice [78]. In this context, Il4ra, a known mediator of IL-4-induced apoptosis in monocytes [101], mast cells [102], hepatocytes [103] and endothelial cells [104], was found to be up-regulated in our study in the hippocampus of aged mice. Accordingly, down-regulation of Il4ra, as occurring with late-onset EE, was proposed as a neuroprotective approach in multiple sclerosis [105]. Age-related up-regulation of Bcl3, as shown here, was also observed in brains suffering from mild cognitive impairment and Alzheimer’s disease [106,107]. Bcl3 was shown to promote TNF-induced apoptosis in hepatocytes [108]; moreover, it is an important inhibitor of NF-kB activity [109]. Bcl3 down-regulation by late-onset EE may therefore mediate molecular pathways that support nerve cell survival and neuronal network plasticity [110]. Furthermore, EE-induced up-regulation of Diexf initiates the proteasome-independent degradation of excessive p53 [111,112], which may further contribute to reducing apoptosis [113] and can eventually lead to the regeneration of damaged and functionally impaired brain tissue as a result of an EE. With Inhba, we identified an important and cross-species regulator of tissue regeneration [114], whose age-related reduced expression is enhanced during late-onset EE in aged mice. 

In summary, our study shows that the brains of old mice that were kept in sensory- and activity-deprived standard housing conditions until 22 months may still have a considerable degree of plasticity. In particular, switching them to an EE that meets the accommodation needs of mice and also figuratively would cover many necessary stimuli in humans, proved to be a promising and non-pharmacological strategy for the treatment of age-related cognitive deficits. Our transcriptome analysis revealed a complex pattern of gene expression changes suggesting that the direction of known age-related molecular signaling cascades mediating dendritic spine stability, senescence, insulin resistance, apoptosis, and regeneration can be positively affected.

## Data Availability

The data presented in this study are available in the article or in the Supplementary Material. The RNA sequencing data discussed in this publication were deposited in NCBI’s Gene Expression Omnibus (Edgar et al., 2002) and are accessible through GEO Series accession numbers GSE111270 and GSE111271. The data sets listed are part of a huge collaboration project called “JenAge” (https://www.ncbi.nlm.nih.gov/gds/?term=jenage (accessed on 28 February 2008)). Therefore, counts and RPKMs were automatically created in the associated data sets in an identical manner for submitting to GEO. These counts and RPKMs were not used in this manuscript. Instead, reads were processed as given in the section “Data analysis”.

## Supplementary Materials

The following supporting information can be downloaded at: https://www.mdpi.com/article/10.3390/cells11233864/s1, Supplementary Table S1: DEGs Age; Supplementary Table S2: DEGs EE; Supplementary Table S3: DEGs Age counter-regulation; Supplementary Table S4:_qPCRValidation; Supplementary Table S5:_effect size.
